# Novel Assays to Distinguish Between Properdin-Dependent and Properdin-Independent C3 Nephritic Factors Provide Insight Into Properdin-Inhibiting Therapy

**DOI:** 10.3389/fimmu.2019.01350

**Published:** 2019-06-17

**Authors:** Marloes A. H. M. Michels, Nicole C. A. J. van de Kar, Ramon M. van den Bos, Thea J. A. M. van der Velden, Sanne A. W. van Kraaij, Sebastian A. Sarlea, Valentina Gracchi, Michiel J. S. Oosterveld, Elena B. Volokhina, Lambertus P. W. J. van den Heuvel

**Affiliations:** ^1^Department of Pediatric Nephrology, Radboud University Medical Center, Radboud Institute for Molecular Life Sciences, Amalia Children's Hospital, Nijmegen, Netherlands; ^2^Department of Pediatric Nephrology, Radboud University Medical Center, Amalia Children's Hospital, Nijmegen, Netherlands; ^3^Crystal and Structural Chemistry, Bijvoet Center for Biomolecular Research, Department of Chemistry, Faculty of Science, Utrecht University, Utrecht, Netherlands; ^4^Department of Laboratory Medicine, Radboud University Medical Center, Nijmegen, Netherlands; ^5^Department of Pediatric Nephrology, University Medical Center Groningen, University of Groningen, Groningen, Netherlands; ^6^Department of Pediatric Nephrology, Emma Children's Hospital, Amsterdam University Medical Center, Amsterdam, Netherlands; ^7^Department of Pediatrics/Pediatric Nephrology and Department of Development and Regeneration, University Hospitals Leuven, Leuven, Belgium

**Keywords:** complement system, alternative pathway, convertase, properdin, C3 nephritic factor, C3 glomerulopathy, therapy, Salp20

## Abstract

C3 glomerulopathy (C3G) is an umbrella classification for severe renal diseases characterized by predominant staining for complement component C3 in the glomeruli. The disease is caused by a dysregulation of the alternative pathway (AP) of the complement system. In more than half of C3G patients C3 nephritic factors (C3NeFs) are found. These autoantibodies bind to the AP C3 convertase, prolonging its activity. C3NeFs can be dependent or independent of the complement regulator properdin for their convertase-stabilizing function. However, studies to determine the properdin-dependency of C3NeFs are rare and not part of routine patient workup. Until recently, only supportive treatments for C3G were available. Complement-directed therapies are now being investigated. We hypothesized that patients with properdin-dependent C3NeFs may benefit from properdin-inhibiting therapy to normalize convertase activity. Therefore, in this study we validated two methods to distinguish between properdin-dependent and properdin-independent C3NeFs. These methods are hemolytic assays for measuring convertase activity and stability in absence of properdin. The first assay assesses convertase stabilization by patient immunoglobulins in properdin-depleted serum. The second assay measures convertase stabilization directly in patient serum supplemented with the properdin-blocking agent Salp20. Blood samples from 13 C3NeF-positive C3G patients were tested. Three patients were found to have properdin-dependent C3NeFs, whereas the C3NeF activity of the other ten patients was independent of properdin. The convertase-stabilizing activity in the samples of the patients with properdin-dependent C3NeFs disappeared in absence of properdin. These data indicate that inhibition of properdin in patients with properdin-dependent C3NeFs can normalize convertase activity and could represent a novel therapy for normalizing AP hyperactivity. Our assays provide a tool for identifying C3G patients who may benefit from properdin-inhibiting therapy and can be incorporated into standard C3G laboratory investigations.

## Introduction

C3 glomerulopathy (C3G) is a recently introduced classification for rare but severe renal diseases characterized by predominant deposition of complement component C3 in glomeruli (1, 2). C3G patients may present with a variety of symptoms, including glomerulonephritis with varying degrees of renal failure, hematuria, hypertension, proteinuria, nephrotic syndrome, and low serum C3. Prognosis is generally poor with a high risk of progression to end-stage renal diseases and a risk of recurrence in kidney transplants of ~50% (3–10). Diagnosis of C3G is made when on kidney biopsy immunofluorescence staining for C3 is ≥2 orders of magnitude higher than for immunoglobulins (Igs) (2, 11, 12). Based on electron microscopy appearance, C3G can be subdivided into dense deposit disease (DDD) and C3 glomerulonephritis (C3GN). DDD is characterized by very dense band-like intramembranous deposits, whereas the deposits found in C3GN are less dense and can show a more variable pattern (2, 12).

The complement system is part of innate immunity and clears pathogens and aberrant host cells from the body (13, 14). Complement can be activated via three pathways: the classical, lectin, and alternative pathway (AP). The complement depositions in C3G are the result of uncontrolled AP activity (12, 15). The AP is constantly active at a low rate by the spontaneous hydrolysis of C3 (tick-over), which allows a quick response to triggers. Hydrolyzed C3 [C3(H_2_O)] is functionally similar to C3b and able to form an initial C3 convertase after it reacts with Factor B and Factor D. The resulting C3(H_2_O)Bb complex cleaves small amounts of C3 into C3a and C3b. Once the system is triggered, an amplification loop leads to full activation of the complement cascade. C3b binds to cell surfaces with its reactive thioester to promote their phagocytosis in a process known as opsonization. Besides, bound C3b can form new C3 convertases (C3bBb) that in turn convert more C3 into C3b. When the C3b density on a surface becomes high enough, C3bBbC3b complexes, better known as C5 convertases, can be formed (16). C5 convertases cleave C5 into its active fragments C5a and C5b, and hereby initiate terminal pathway activity. The end product is a pore-forming, lytic C5b-9 complex, also known as the membrane attack complex.

In healthy individuals, the AP is strictly regulated. Circulating factors in blood, such as Factor H (FH) and Factor I (FI), and membrane-bound inhibitory proteins act together to protect from excessive AP activation and AP attack on healthy cells. Inhibitors of C3 convertase activity promote the decay of the convertase complex or act as cofactors for FI, which enzymatically inactivates C3b resulting in C3b breakdown fragments (14, 17). It is these C3 breakdown fragments that are typically found in the glomeruli of C3G patients (18). There is also a positive regulator that promotes instead of inhibits C3 convertase activity: properdin, a glycoprotein mainly synthesized by leukocytes (19, 20). Properdin stabilizes the C3bBb complex by forming C3bBbP, which enhances its half-life 5–10 fold (21). The exact function of properdin in the C5 convertase is not known yet (20).

When the sophisticated balance of complement activation and inhibition is disturbed, damage can occur to healthy host tissues. The glomeruli are thought to be especially vulnerable to overactive complement activity (22, 23). In patients with C3G, several pathogenic causes leading to an overactive AP can be found. Around 20% of patients has mutations in genes encoding for complement inhibitory proteins (loss-of-function mutations) or activating components (gain-of-function mutations) (5, 8, 15). A more common finding in C3G patients are C3 nephritic factors (C3NeFs). These autoantibodies stabilize the C3 convertase complex and prolong its activity (24). In contrast to properdin, C3NeFs are not part of normal human complement homeostasis. They are found in ~40–50% of C3GN patients and in ~80% of DDD patients (5, 8, 25–27).

C3NeFs are functionally heterogeneous. The autoantibodies bind to neoepitopes on the C3bBb(P) complex to inhibit the intrinsic, spontaneous and extrinsic, accelerated decay mediated by complement regulators to varying degrees (28–32). Besides, studies dating back to the late 80s/90s have reported that C3NeFs may be dependent or independent of properdin for their ability to stabilize convertases (33, 34). Several recent studies have confirmed the presence of these two types of C3NeF (4, 27, 31).

At the moment, therapy for C3G is mainly supportive and entails anti-proteinuric and immunosuppressive medication (10, 12). In recent times, the focus has shifted more and more toward targeting of the complement system (35). Eculizumab, a humanized anti-C5 antibody, was proven very efficacious in patients with atypical hemolytic uremic syndrome (36), which is also a renal disease strongly associated with uncontrolled AP activity. Nonetheless, C3G patients have shown an inconsistent response to this drug (37). Since the clinical features of C3G may derive predominantly from C3 activation, C3G patients may benefit from blocking C3 and/or C3 convertase activity (38). Therefore, there is increasing interest in inhibition of AP complement activation at an early phase, such as inhibiting C3, Factor B or Factor D (35, 39), or non-canonical targets such as renin, which also has recently been shown to have C3-cleaving capacities (40).

We hypothesized that C3G patients with properdin-dependent C3NeFs might benefit from therapies targeting properdin, leading to normalization of C3 convertase activity. In this study, we established methods to distinguish between properdin-dependent and properdin-independent C3NeFs and subsequently investigated whether convertase stabilization could be normalized by properdin inhibition in patients with properdin-dependent C3NeFs.

## Materials and Methods

### Human Serum and EDTA-Plasma Samples

Serum and ethylenediamine-tetraacetic acid (EDTA)-plasma samples were obtained from patients referred to the Radboudumc and from healthy controls. Exclusion criteria for healthy controls were fever, bacterial and/or viral infections in the preceding 2 weeks, chronic illness, inherited or acquired immune disorders, and immunosuppressive medication. The study was carried out in accordance with the recommendations of the appropriate version of the Declaration of Helsinki. Samples were processed according to protocol as previously described (41). For control material, pooled normal human serum (NHS) and pooled normal human EDTA-plasma (NHP) were produced from samples of 20 healthy volunteers. In addition, heat-inactivated NHS (hi-NHS) was prepared by incubating NHS for 30 min. at 56°C. Properdin-depleted (ΔP-NHS; A339) and C3-depleted normal human serum (ΔC3-NHS; A314) were purchased from Complement Technology (Tyler, TX, USA).

### Quantification of Complement Proteins and Activation Products

Serum C3 levels were measured by nephelometry following the standardized diagnostic procedure of the Radboudumc using IMMAGE^®^ Immunochemistry Systems (Beckman Coulter, Brea, CA, USA). Sandwich enzyme-linked immune sorbent assays (ELISAs) were used to quantify all other complement (regulatory) proteins. Except for the properdin ELISA, all protocols are currently operational in routine diagnostics at the Radboudumc. C5 levels were measured in human serum or EDTA-plasma using goat polyclonal antiserum to human C5 (A306; Quidel, San Diego, CA, USA), combined with a mouse monoclonal anti-human C5 antibody (A217; Quidel) for detection. FH and FI were quantified in serum or EDTA-plasma samples. FH was detected using a polyclonal goat anti-human FH antibody (A312; Quidel), followed by a mouse monoclonal anti-human FH antibody (A229; Quidel). FI was quantified using a polyclonal sheep anti-human FI antibody (LN1301932; LabNed, Amstelveen, the Netherlands) and subsequently detected using a mouse monoclonal anti-human FI antibody (OX-21; ProSci, Poway, CA, USA). Plate readout was similar for these assays, namely using polyclonal goat anti-mouse antibodies conjugated to horseradish peroxidase (P0447; Dako, Glostrup, Denmark) followed by the substrate o-phenylenediamine dihydrochloride. Results were calculated based on calibration lines produced by the commercially obtained purified human protein standards C5 (204888; Calbiochem), FH (341274; Calbiochem), and FI (A138, Complement Technology). The activation markers C3bBbP, C3bc, and C5b-9 were measured in EDTA-plasma by ELISA as previously described (42, 43). The ELISA for properdin detection in serum was set up and optimized based on previously described protocol (44). In brief, a mouse monoclonal anti-human properdin antibody (A233; Quidel) was used as the capture antibody and for detection a polyclonal rabbit anti-human properdin antibody labeled with digoxigenin (a kind gift of Prof. C. van Kooten, Leiden University Medical Center). After addition of an anti-digoxigenin antibody conjugated to horseradish peroxidase (Roche, Mannheim, Germany), the plate was developed with tetramethylbenzidine and results were calculated based on a calibration line produced by an NHS standard that was calibrated against the described serum standard (44).

### Salp20 Production and Purification

A DNA fragment encoding *Ixodes scapularis* Salp20 [UniProtKB: Q95WZ1 (residue 22–183)] was amplified by PCR from Salp20 synthetic DNA optimized for mammalian expression (GeneART ThermoFisher), and ligated into BamHI-NotI sites of vector pUPE106.03 (U-Protein Express BV, Utrecht, the Netherlands). The expressed protein contained a cystatin secretion signal peptide, an N-terminal (His6)GlySer-tag and an C-terminal Ala3 cloning artifact due to the NotI restriction site. The construct was transiently expressed in N-acetylglucosaminyltranferase I-deficient (GnTI-) Epstein-Barr virus nuclear antigen I(EBNA1)-expressing HEK293 cells cultured in suspension (U-Protein Express BV, Utrecht, the Netherlands). Secreted Salp20 was captured by incubating culture medium with Ni-Sepharose excel beads (GE Healthcare) at 4°C for 2 h, followed by washing with 25 mM HEPES pH 7.8, 500 mM NaCl, 15 mM imidazole. After elution using the washing buffer supplemented with 250 mM imidazole the sample was further purified by gel-filtration using a Superdex 200 increase 10/300 GL column (GE Healthcare) equilibrated in 25 mM HEPES pH 7.8, 150 mM NaCl. Salp20 was concentrated to 8.4 mg/ml by centrifugation using a 5-kDa cut-off concentrator before plunge freezing in liquid nitrogen and storage at −80°C.

### Ig Purification

Purified Ig fractions were obtained from the EDTA-plasma samples of patients P1 to P6 and from NHP as previously described (41). In short, Igs were isolated using a protein A/G affinity chromatography column (Thermo Fisher Scientific, Waltham, MA, USA). Ig fractions were subsequently dialyzed against phosphate buffered saline and concentrated to the original plasma sample volume using a concentrator with a 10 kDa molecular weight cut-off. The Ig concentrations were measured using NanoDrop Spectrophotometer (Thermo Fisher Scientific) and yielded: 4.7 mg/ml (P1), 2.1 mg/ml (P2), 9.7 mg/ml (P3), 10.2 mg/ml (P4), 8.6 mg/ml (P5), 12.5 mg/ml (P6), and 10.7 mg/ml (NHP). The Ig fractions were not contaminated with properdin as tested in the ELISA above.

### Convertase Activity Assays

AP convertase activity assays are two-step hemolytic assays in which the assembly of convertases is separated from C5b-9 formation and hemolysis using a C5-blocking agent. These assays were performed according to a previously described protocol (41) but with adaptations in the first step of the assay for convertase assembly. Rabbit erythrocyte (RbE) working suspensions were prepared by washing the RbE in a magnesium-ethylene glycol tetraacetic acid (Mg-EGTA) buffer (2.03 mM veronal buffer, pH 7.4, 10 mM EGTA, 7 mM MgCl_2_, 0.083% gelatin, 115 mM D-glucose, and 60 mM NaCl) followed by calibration to standardize the number of erythrocytes in each experiment. In all assays, 10 μl of prepared RbE were mixed with 20 μl of 150 nM of the C5 inhibitor eculizumab diluted in Mg-EGTA. Subsequently, at different time points 20 μl of human test serum, i.e., NHS, ΔP-NHS, patient serum or ΔC3-NHS, diluted in Mg-EGTA to a concentration of 9.5 or 12.5%, was added for the convertase assembly at 37°C. Alternatively, the test serum was supplemented with Ig fractions in a 1:1 (standard) or 1:3 volume ratio, or with purified properdin (A139, Complement Technology), Salp20, and/or C3 (A113, Complement Technology) to obtain final concentrations as indicated in figure legends. These protein concentrations are corrected for the used serum percentage, i.e., indicating the concentration per volume of undiluted serum. Patient sera were always tested mixed with an equal volume of NHS to compensate for possible low C3 levels (41). As a model for patient serum with low C3, ΔC3-NHS was treated similarly. After convertase assembly in this first step, erythrocytes were washed with cold EDTA-gelatin veronal buffer (EDTA-GVB; 4.41 mM veronal buffer, 0.1% gelatin, 130 mM NaCl, pH 7.4) to wash away excessive C5-inhibitor and unbound complement components. The second step of the assay, in which the recovered erythrocytes were overlaid with guinea pig serum diluted in EDTA-GVB as a source of C5b-9 components for generating hemolysis, was not subjected to any changes in this study. Hemolysis levels are given as percentage of full lysis of erythrocytes in water, and hi-NHS served as a negative control in all assays.

## Results

### Patient Characteristics and Complement Profiles

For this study, 13 pediatric patients (age range 3–17 years) who were referred to the Radboudumc because of a (suspicion of) C3G diagnosis were selected based on a positive test for prolonged convertase activity, i.e., C3NeF activity, in diagnostic settings (41), and based on the availability of sufficient and appropriate serum and plasma samples taken in the diagnostic phase (Table 1). Subdivision into C3GN or DDD was based on the renal pathology report following the guidelines described in the consensus report of the first C3G Meeting (2). One patient did not meet the C3G criteria and was diagnosed with immune complex-mediated membranoproliferative glomerulonephritis (IC-MPGN). Genetic complement analysis and the analysis of complement parameters in serum/plasma were performed in all patients (Table 1). Complement activation markers and levels of complement (regulatory) proteins showed strong variations among patients. All samples taken for these complement investigations, including the testing for properdin dependency of C3NeFs, were obtained during the initial diagnostic phase at presentation (P2, P4, P5, P6, P8, P9, and P11) or during a later stage of disease in which patient samples remained positive for C3NeF activity (P1, P3, P7, P10, P12, and P13). Patients P1, P3, P7, P10, and P13 continued to show signs of ongoing complement activation, i.e., low serum C3 levels and elevated complement activation products, despite of normal renal function. Interestingly, the levels of C3, C5, and properdin of P12 were decreased at initial presentation (combined with elevated C3bBbP and TCC), but the complement consumption and renal function normalized after 1 year. Data shown for this patient in Table 1 belong to this partial remission phase in which C3NeF activity remained present, since not enough patient material from acute phase was available. Due to the absence of EDTA-plasma material from the acute phase of P8, the reported activation markers C3bBbP, C3bc, and TCC for this patient are from a later stage of disease in which there was ongoing C3 activation. Patient P2 developed end-stage renal disease within the first 3 months after presentation and received hemodialysis.

**Table 1:** Complement investigations in C3NeF-positive C3G patients.

**Patient**	**Gender (M/F)**	**Age at time of first presentation (year)**	**Age at time of study (year)**	**Diagnosis**	**Genetic aberrations**	**Properdin-dependency of C3NeF**	**C3 (mg/L) [700–1500]**	**C5 (μg/ml) [42-93]**	**Properdin (μg/ml) [11.0–28.0]**	**C3bBbP (CAU) [<12]**	**C3bc (CAU) [<15]**	**C5b-9(CAU) [<0.5]**	**Factor H (μg/ml) [122–315]**	**Factor I (μg/ml)[22-41]**
**(A) PATIENTS OF WHICH BOTH THE Ig FRACTIONS AND SERA ARE TESTED FOR PROPERDIN-DEPENDENCY OF C3NeF**														
P1	F	8	9	C3GN	*CFHR5* c.542G>C (p.R181T)	Independent	***130***	***23.3***	17.1	***23.1***	10.4	***8.6***	***400***	34
P2	M	6	6	DDD		Independent	***400***	***34.7***	15.6	6.74	7.3	***1.3***	***328***	***50***
P3	M	6	17	DDD		Independent	***90***	***41.4***	14.8	***21.4***	***24.9***	***1.0***	259	25
P4	M	15	15	C3GN		Dependent	***70***	***20.7***	***8.2***	***29.1***	***22.8***	***7.8***	311	35
P5	M	5	5	DDD		Independent	***110***	61.1	17.3	7.24	***16.3***	***1.9***	184	36
P6	F	5	5	C3GN		Independent	1190	52.2	13.0	***23.7***	9.2	0.5	189	41
**(B) PATIENTS OF WHICH THE SERA ARE TESTED FOR PROPERDIN-DEPENDENCY OF C3NeF**														
P7	M	6	11	DDD		Independent	***100***	48.4	***31.8***	<2	8.7	0.5	264	22
P8	F	7	7	DDD		Independent	***141***	58.5	***31.7***	***12.4***	***27.1***	0.3	252	26
P9	F	16	16	DDD	*DGKE* c.851G>A (p.G284E)	Independent	**252**	***41.2***	22.7	3.2	4.4	0.4	***116***	***21***
P10	F	16	17	C3GN	*C3* c.691A>C (p.S231R)	Dependent	***91***	***23.1***	***9.4***	***55.8***	***16.3***	***8.0***	210	***21***
P11	F	13	13	DDD	*CFHR1/CFHR4 deletion*	Independent	***194***	***35.0***	21.6	***26.5***	8.7	***4.9***	141	***20***
P12	M	2	3	C3GN		Independent	878	65.7	20.9	***15.4***	6.9	0.4	309	***50***
P13	M	11	15	IC-MPGN		Dependent	***60***	***5.0***	11.3	***33.1***	***26.3***	***3.5***	167	***20***

*Complement investigations were performed at the time of study. Reference ranges based on healthy controls are indicated between square brackets, and values outside this range are indicated in bold italic. All patients tested negative for the presence of autoantibodies against Factor H. Indicated genetic aberrations are heterozygous variations. C3GN, C3 glomerulonephritis; DDD, dense deposit disease; IC-MPGN, immune complex-mediated membranoproliferative glomerulonephritis; CFHR5, complement factor H-related 5; C3NeF, C3 nephritic factor*.

### Two Novel Methods to Distinguish Between Properdin-Dependent and Properdin-Independent C3NeFs

**Figure 1:**
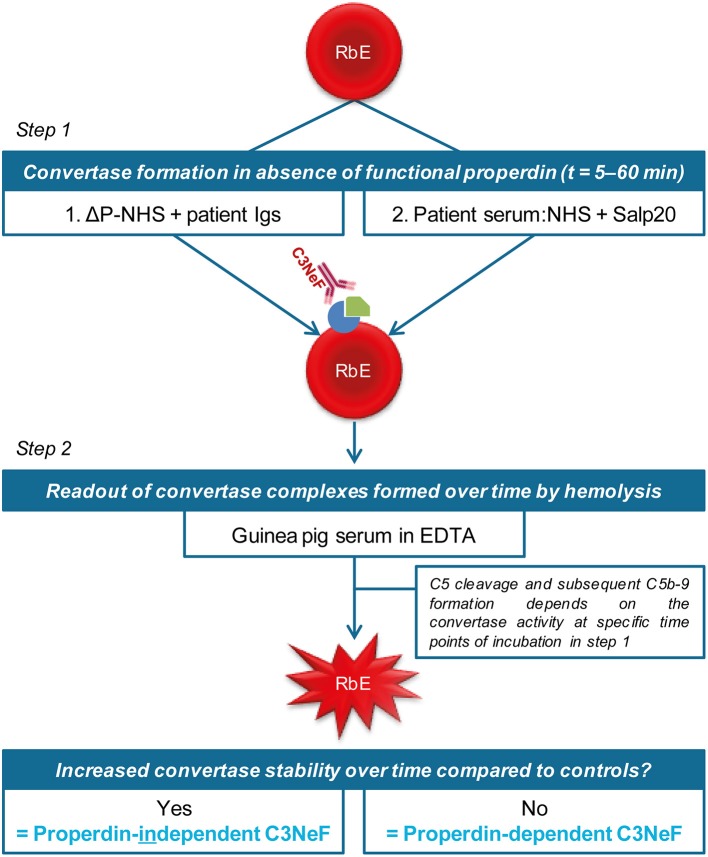
Two approaches to distinguish between properdin-dependent and properdin-independent C3 nephritic factors (C3NeFs). In the first approach, rabbit erythrocytes (RbE) are incubated with properdin-depleted normal human serum (ΔP-NHS) substituted with an equal volume of patient immunoglobulins (Igs). In the second approach, properdin function is eliminated by adding the properdin-blocking tick protein Salp20 to patient serum, that is always mixed 1:1 with pooled normal human serum (NHS) to compensate for possible low C3 levels. All incubations in the first step take place under C5 inhibition by eculizumab to prevent complement activation up to the level of the C5b-9 complex formation and subsequent hemolysis. After a washing step, guinea pig serum in presence of EDTA is added in the second step of the assay as a source of C5b-9 components to read out the activity of preformed convertase complexes of the first step. Hemolysis is measured over time to form convertase activity profiles. Prolonged convertase activity in absence of properdin indicates increased convertase stabilization by properdin-independent C3NeFs, whereas normal convertase stability indicates that the C3NeFs are properdin-dependent.

We previously validated a hemolytic assay to monitor convertase activity over time in human serum. This method enabled the detection of factors causing increased convertase stability such as C3NeFs in patient serum (41). In this assay, the stage of convertase formation by test sera (step 1) is separated from the standardized stage of C5b-9 formation and hemolysis (step 2) by using the C5 inhibitor eculizumab. We assume that all important events influencing C3 convertase activity are also reflected in the C5 convertase activity that eventually generates the readout. In this study, we aimed to modify the existing assay to distinguish between properdin-dependent and properdin-independent C3NeFs. For this purpose, two approaches were designed in which properdin was eliminated during the first step of convertase assembly and decay in presence of C3NeFs (Figure 1). The first method assesses the ability of C3NeF-positive patient Ig fractions to stabilize convertases formed out of ΔP-NHS. In the second assay, properdin is blocked directly in serum using the properdin-blocking tick protein Salp20 (45) to assess the ability of the C3NeFs contained in serum to cause convertase stabilization. Only samples with properdin-independent C3NeFs test positive in these assays.

**Figure 2:**
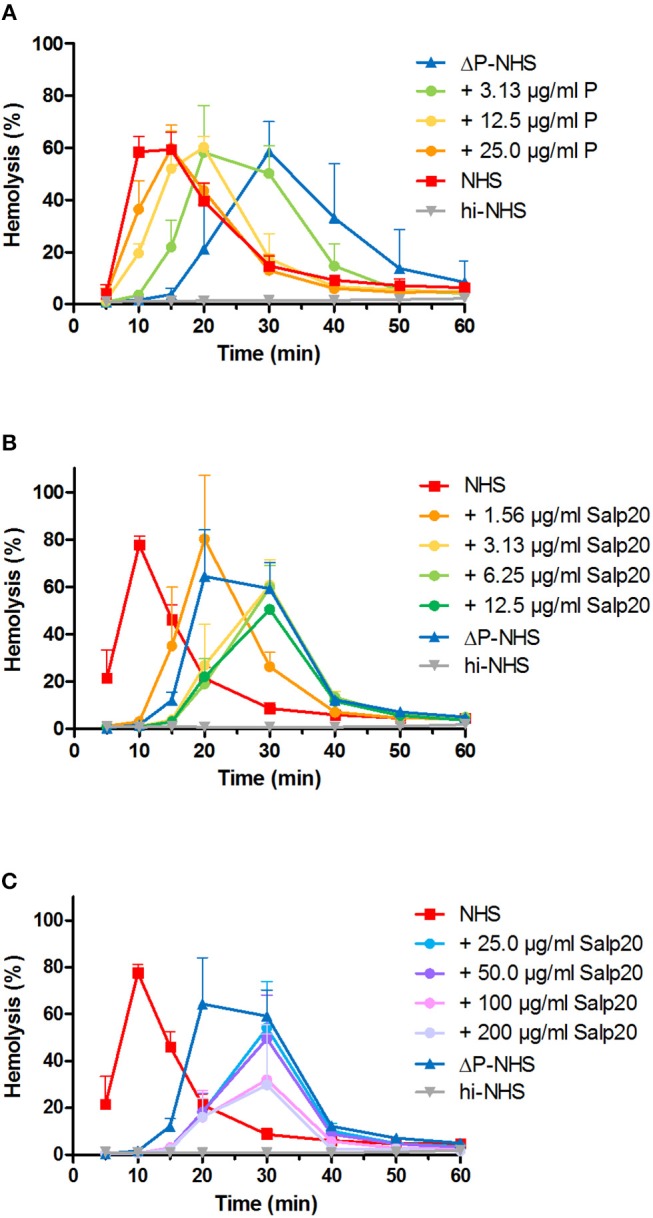
Properdin depletion or blockage in normal human serum results in delayed maximal convertase activity. **(A)** Rabbit erythrocytes were incubated with properdin-depleted normal human serum (ΔP-NHS) and increasing concentrations of purified properdin (P). **(B,C)** Alternatively, erythrocytes were incubated with pooled normal human serum (NHS) and increasing concentrations of Salp20. Protein concentrations indicated are corrected for the used serum percentages of 3.8 **(A)** and 5.0% **(B,C)**. Error bars indicate standard deviations of the mean obtained from three independent experiments. hi-NHS, heat-inactivated NHS.

We first investigated the effect of absence of properdin in the convertase activity assay using ΔP-NHS. We confirmed by ELISA that no residual properdin was detectable in this commercially obtained ΔP-NHS. Whereas maximal convertase activity (t_max_) induced by NHS is generally reached after 10–15 min of incubation, the t_max_ of ΔP-NHS was reached after 30 min (Figure 2A). Reconstitution of ΔP-NHS with purified properdin restored the convertase activity dose-dependently. A normal physiological concentration of 25 μg/ml properdin was able to completely bring back t_max_ to 15 min as in NHS.

Subsequently, we examined the effect of Salp20 on convertase formation and decay in the convertase activity assay. The addition of at least 3.13 μg/ml Salp20 (170 nM) to NHS resulted in convertase activity profiles similar to those of ΔP-NHS and were characterized by a delayed t_max_ of 30 min (Figure 2B). This supports that effective inhibition of properdin in NHS was possible using Salp20. If the properdin blocker was added in much higher concentrations, i.e., up to 100 or 200 μg/ml (up to 11 μM), a decrease in maximal hemolysis was observed (Figure 2C). Therefore, 6.25 μg/ml (340 nM) was chosen as the optimal concentration for properdin blockage in serum in following experiments. Altogether, both approaches showed consistent differences between the convertase activity profiles of serum in absence and presence of functional properdin.

### Detection of Properdin-Dependent C3NeFs in Patient Ig Fractions

**Figure 3:**
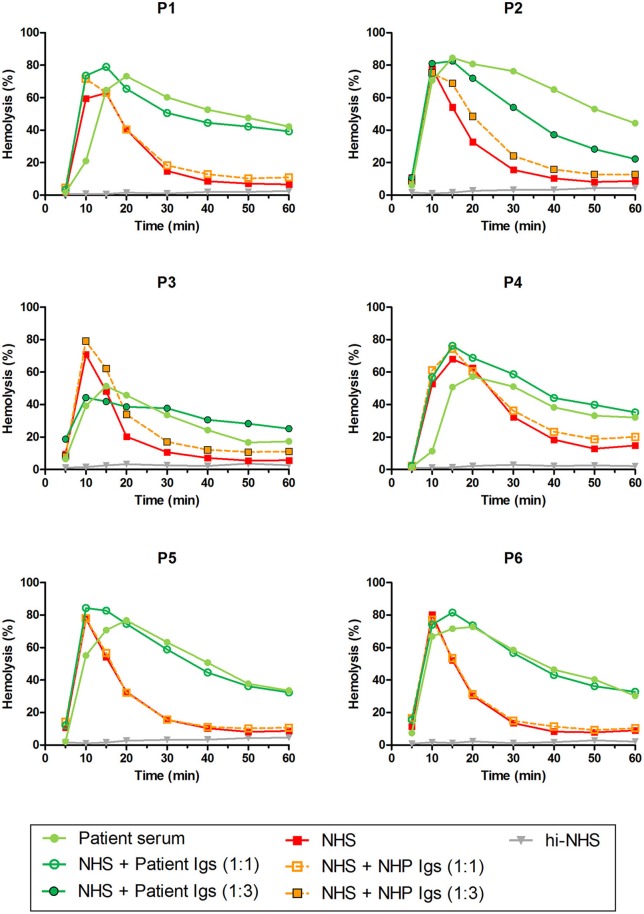
C3NeF activity in patient sera and patient immunoglobulins (Igs). Rabbit erythrocytes were incubated with pooled normal human serum (NHS) mixed with an equal volume of serum of patients P1, P2, P3, P4, and P6 or EDTA-plasma of P5 to a final concentration of 5%. Alternatively, erythrocytes were incubated with NHS mixed with an equal volume (1:1; P1, P4, P5, and P6) or 3-fold volume (1:3; P2 and P3) of purified Igs derived from patient samples or with control Igs derived from pooled normal human EDTA-plasma (NHP). Representative data of at least two independent experiments are shown. hi-NHS, heat-inactivated NHS.

**Figure 4:**
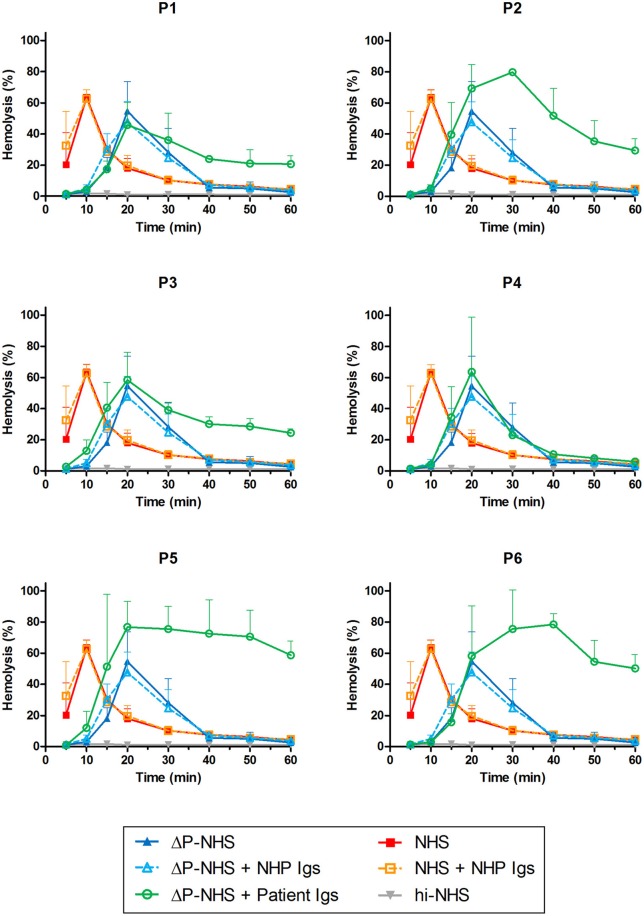
Detection of properdin-dependent and properdin-independent C3NeFs in C3G patients using properdin-depleted normal human serum (ΔP-NHS) and patient immunoglobulins (Igs). Rabbit erythrocytes were incubated with 5% ΔP-NHS mixed with an equal volume of purified Igs from patients P1, P2, P3, P4, P5, and P6 or with control Igs derived from pooled normal human EDTA-plasma (NHP). Error bars indicate standard deviations of the mean obtained from three independent experiments. NHS, pooled normal human serum; hi-NHS, heat-inactivated NHS.

Patients P1 to P6 all showed prolonged convertase activity in the assay under default conditions with properdin present. We confirmed that this prolonged activity was caused by convertase-stabilizing autoantibodies, i.e., C3NeFs, by adding the purified Ig fractions of the patient samples to NHS (Figure 3). We subsequently tested the C3NeF activity of patient Igs in ΔP-NHS. Five out of the six tested C3NeF-containing fractions, namely of P1, P2, P3, P5, and P6, showed significant prolongation of convertase activity when added to ΔP-NHS (Figure 4). In contrast, the addition of the Igs of P4 to ΔP-NHS did not induce any change in convertase stability and resulted in a profile similar to ΔP-NHS incubated with control Igs (NHP Igs). Thus, the C3NeF activity observed in P1, P2, P3, P5, and P6 was independent of properdin, whereas the stabilizing activity of the C3NeFs of P4 was likely dependent on presence of properdin during convertase formation.

### Detection of Properdin-Dependent C3NeFs in Patient Serum

**Figure 5:**
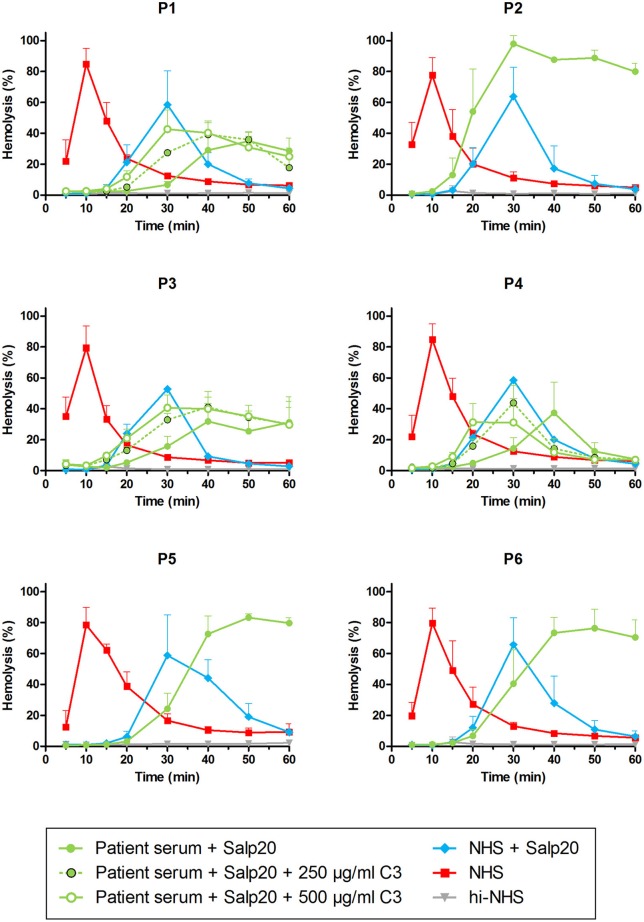
Detection of properdin-dependent and properdin-independent C3NeFs in C3G patients using patient serum and the properdin inhibitor Salp20. Rabbit erythrocytes were incubated with pooled normal human serum (NHS) mixed with an equal volume of serum of patients P1, P2, P3, P4, and P6 or EDTA-plasma of P5 to a final concentration of 5% and in presence of 6.25 μg/ml Salp20. Additionally, purified C3 was added for the samples of P1, P3, and P4, according to the concentrations indicated, which are corrected for the used serum percentage. Error bars indicate standard deviations of the mean obtained from three independent experiments. ΔP-NHS, properdin-depleted normal human serum; hi-NHS, heat-inactivated NHS.

As an alternative approach, we aimed to assess the properdin dependency of C3NeFs directly in patient serum by blocking properdin with Salp20. Not enough serum was available for P5, so EDTA-plasma was used. This material was previously shown to be compatible with the assay (41). The C3NeFs contained in the serum or plasma of patients P2, P5, and P6 clearly retained their ability to prolong the convertase activity in the presence of Salp20 (Figure 5). The convertase activity profiles obtained from sera of patients P1, P3, and P4 were more difficult to interpret for prolonged convertase activity, since hemolysis levels were lower and maximal activity was reached at later time points compared to NHS treated with Salp20.

**Figure 6:**
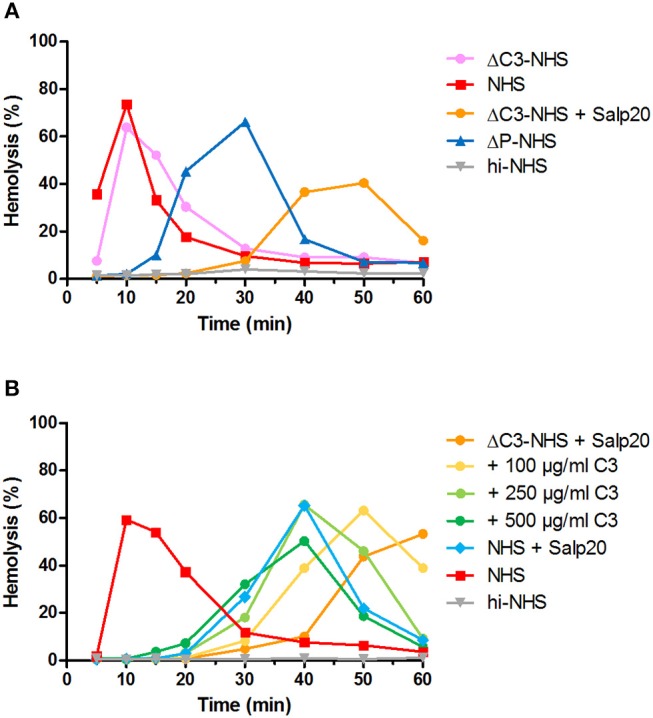
Effect of low serum C3 concentration on the convertase activity profile of serum treated with Salp20. **(A)** Rabbit erythrocytes were incubated with C3-depleted normal human serum (ΔC3-NHS) mixed with an equal volume of pooled normal human serum (NHS) in absence or presence of 6.25 μg/ml Salp20. **(B)** Alternatively, ΔC3-NHS samples treated with Salp20 were supplemented with increasing concentrations of purified C3. Concentrations indicated are corrected for the used serum percentages of 5.0 **(A)** and 3.8% **(B)**. Representative data of at least two independent experiments are shown. ΔP-NHS, properdin-depleted normal human serum; hi-NHS, heat-inactivated NHS.

We hypothesized that this could be due to inadequate compensation for the low serum C3 levels in the samples under these particular properdin-lacking conditions. Using ΔC3-NHS as a substitute for patient serum with very low C3 we confirmed this hypothesis. Like patient sera, ΔC3-NHS was tested in a 1:1 ratio with NHS, resulting in half of the normal C3 levels. This compensation is adequate in conditions in which properdin is present (41). However, in serum conditions with properdin absent or inhibited by Salp20, reduced C3 levels by half significantly altered the activity profile characterized by a t_max_ reached at 50 min (Figure 6A). Such activity profile does not allow analysis for prolongation of convertase activity after t_max_ is reached. The addition of C3 to reach physiological levels could restore the activity profile and t_max_ dose-dependently (Figure 6B).

Thus, samples P1, P3, and P4 were supplemented with 250 and 500 μg/ml purified C3 to shift hemolysis peaks to earlier time points comparable to those of samples with normal C3. This recovered the experimental time window to assess the activity profiles and clearly revealed that the C3NeFs of P1 and P3 still caused convertase stabilization in absence of functional properdin, whereas those of P4 did not (Figure 5). As a control, the addition of C3 to P2 and P5 only resulted in a slightly faster generation of t_max_, but it did not alter the prolonged convertase activity present in these patients (Supplementary Figure 1). These results obtained with the Salp20 method correspond to those generated with ΔP-NHS and Igs and further support the properdin-dependency of the C3NeFs of P4.

### Properdin Restores the Convertase-Stabilizing Activity of Properdin-Dependent C3NeFs in a C3G Patient

**Figure 7:**
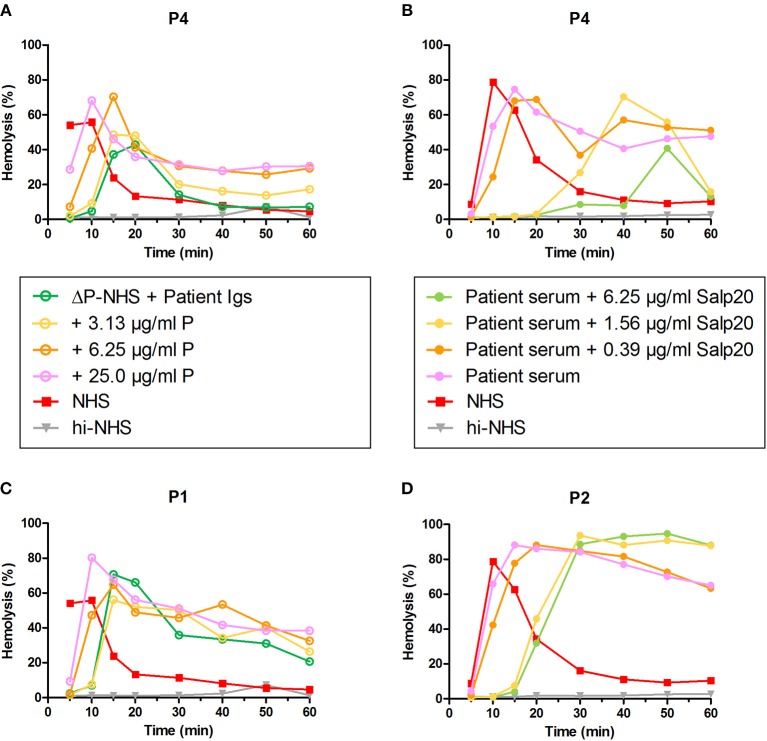
Convertase-stabilizing activity of properdin-dependent but not properdin-independent C3NeFs is restored in presence of functional properdin. Patient immunoglobulins (Igs) of P4 **(A)** and P1 **(C)** were incubated with properdin-depleted normal human serum (ΔP-NHS), and were reconstituted with increasing concentrations of purified properdin (P). Alternatively, Salp20 was titrated into the patient sera of patient P4 **(B)** and P2 **(D)**, that were tested mixed with an equal volume of pooled normal human serum (NHS). Protein concentrations indicated are corrected for the used serum percentage of 5%. Representative data of at least two independent experiments are shown. hi-NHS, heat-inactivated NHS.

To confirm the properdin-dependency of the C3NeFs of P4, functional properdin was restored in both assays by either reconstituting the ΔP-NHS with purified properdin or by titrating Salp20 to lower, ineffective concentrations in the patient serum. Convertase stabilization by P4 Igs completely returned upon addition of purified properdin in concentrations of 6.25 μg/ml or higher (Figure 7A). In a similar way, titration of Salp20 to concentrations below 6.25 μg/ml restored the convertase stabilization by C3NeFs in the serum of P4 (Figure 7B). Availability of functional properdin also resulted in a shift of t_max_ to earlier time points, as we previously observed in Figure 2. Except for this peak shift, properdin reconstitution in the samples of P1 and P2, containing properdin-independent C3NeFs, did not affect the convertase stabilization (Figures 7C,D). In conclusion, these experiments support our previous findings that the convertase-stabilizing activity by P4 C3NeFs was dependent on properdin.

### Screening for Properdin-Dependent C3NeFs in Patient Sera Using the Salp20 Method

**Figure 8:**
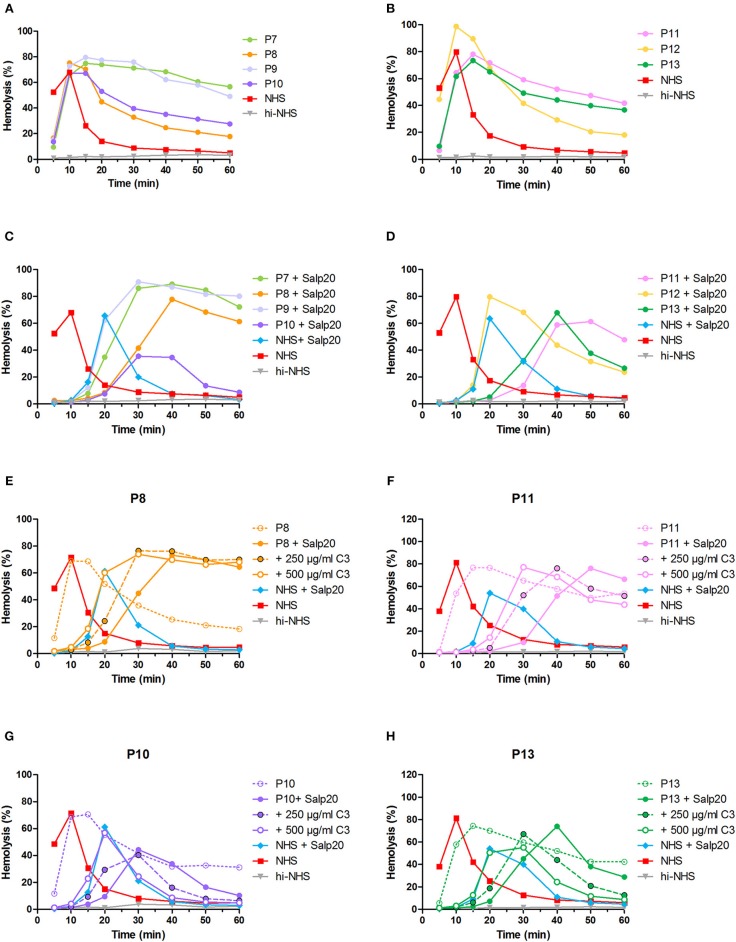
Screening for properdin-dependent C3NeFs in patient sera using the Salp20 method. Convertase activity was assessed in the serum of patients P7–P13 mixed with an equal volume of pooled normal human serum (NHS) to a final percentage of 3.8% **(A,B)**. Convertase activity in absence of properdin was assessed by adding 6.25 μg/ml Salp20 to these samples **(C,D)**. For P8, P10, P11, and P13, convertase activity was also assessed after compensation for C3 by adding 250 and 500 μg/ml purified C3 to the samples **(E–H)**. All concentrations indicated have been corrected for the used serum percentage of 3.8%. Representative data of at least two independent experiments are shown. hi-NHS, heat-inactivated NHS.

After the validation of our methods to detect properdin-dependent C3NeFs in six patients, we then applied the method using Salp20 for C3NeF characterization in seven other patient sera that we tested positive for C3NeF (Figures 8A,B). We chose this Salp20 method for the screening as it is less time consuming (no Ig purification) and it requires less patient material. Regardless of the C3 levels of these samples, we first decided to test all samples with Salp20 to block properdin but without the addition of extra C3 (Figures 8C,D). In 4 out of 7 samples, we observed that t_max_ was generated at later time points compared to NHS treated with Salp20, thereby reducing the time window in which convertase activity could be assessed. Hence, these samples, belonging to P8, P10, P11, and P13, were selected for C3 compensation with 250 and 500 μg/ml C3 (Figures 8E–H). This approach enabled a clear and reliable assessment of the convertase activity profiles under properdin blockade. The sera of patients P10 and P13 were completely unable to cause prolonged convertase activity when properdin was blocked (Figures 8G,H). Thus, the C3NeF activity in P10 and P13 was properdin-dependent, whereas the convertase-stabilizing activity in the other five patients was detected independent of properdin.

In total, we analyzed 13 pediatric patients in this study, three of which carry properdin-dependent C3NeFs.

## Discussion

Patients with C3G have a variable disease course and can present with heterogeneous genetic backgrounds and complement activation profiles. This complicates therapeutic choices. The careful characterization of different types of C3NeF, such as properdin-dependent and properdin-independent C3NeFs, may enable a better understanding of the underlying complement problems and provide better patient stratification. More importantly, it might lead to new therapeutic options for C3G patients. This also holds true for patients diagnosed with IC-MPGN, in which C3NeFs are also common (5). We hypothesized that inhibiting properdin can compensate for the convertase-stabilizing effect of properdin-dependent C3NeFs. In this study, we optimized two simple and reliable methods to distinguish between properdin-dependent and properdin-independent C3NeFs. Of the 12 C3G patients and 1 IC-MPGN patient we tested, 3 patients were found positive for having properdin-dependent C3NeFs. For these patients, we showed *in vitro* proof-of-concept that properdin inhibition in serum could normalize the convertase activity.

The described assays assess the ability of C3NeFs to stabilize convertases formed out of serum in which properdin is eliminated using ΔP-NHS or the properdin-inhibiting protein Salp20 (Figure 1). Hereby, they allow the strict distinction between C3NeFs that do or do not function in absence of properdin in a serum environment. Our approaches resemble those initially developed in the late 80s/90s: they are based on removing properdin from the default assay conditions (33, 34). In contrast, most of the recently published studies determined the properdin-dependency of C3NeFs by adding properdin to the default conditions (4, 27, 31). These studies showed that some C3NeFs could not be detected when the stabilization of the C3bBb complex was assessed but could be detected when C3bBbP stabilization was investigated (4, 27, 31). The convertase complexes in these studies were formed using purified components on the platform of sheep erythrocytes or an ELISA-plate. The advantage of the assays described in our study is that convertase formation takes place in a whole serum environment, which more closely resembles physiological conditions regarding convertase formation and regulation. Therefore, they are more likely to reveal physiologically relevant stabilizing factors. Nonetheless, in the current setting, a standardized serum source is used as well as a rabbit erythrocyte platform, which is not comparable to the glomerular structures in terms of surface binding places for complement regulators and complement inhibitors expressed. Future research may focus on further assay optimization using human (glomerular) cells.

The two validated assays presented here have their own benefits and drawbacks. The assay that tests the capability of patient Igs to stabilize convertases formed out of ΔP-NHS has the advantage that it directly confirms the autoantibody nature of the convertase-stabilizing factor present in the patient. In addition, it uses a standardized commercial source for convertase formation and thereby reduces assay variability. On the other hand, this approach requires Ig purification which is a relatively time-consuming step requiring a high volume of patient material. The second assay, in which the properdin dependency is assessed directly in patient serum mixed with NHS in presence of Salp20, has the advantage that it resembles the physiological situation in the patient more closely. Also, there is no need for Ig purification (making the assay less time consuming) and the amount of serum needed is minimal. For this reason, we chose this method to screen for other properdin-dependent C3NeFs in additional patient sera. However, we found that for some serum samples with very low C3 levels and late onset of t_max_, compensation with purified C3 was needed to create an experimental time window allowing the reliable assessment of the convertase activity profiles (Figures 5, 8). This indicates that experimental conditions should be well-optimized and well-regulated. Both assays can be easily incorporated in routine laboratories with expertise in complement methods to be used for patient screening.

Some studies have reported that properdin-dependency of C3NeFs correlates with complement biomarker profiles in various patient groups. Properdin-dependent C3NeFs were associated with C5 convertase dysregulation and activation of the terminal pathway, whereas properdin-independent C3NeFs were predominantly associated with C3 convertase dysregulation (4, 33, 34). In our study with a small number of patients, we did not observe such correlations. Decreased C3 and C5 levels and increased C5b-9 levels were observed with both types of C3NeF (Table 1). Future research with larger patient numbers may shed light on the mechanism of action of different circulating C3NeF types and the association with complement (activation) markers.

During assay development, we gained important insights in the dynamic process of convertase assembly and decay in presence or absence of properdin. Using the convertase activity assays, we showed that in absence of properdin, convertase readout was obtained after a much longer incubation time (Figure 2). These results indicate that in absence of this convertase-stabilizing protein, convertase formation was less efficient. The process could be easily restored upon addition of purified properdin in a dose-dependent manner. Thus, our assay can be used as a platform for future experiments to study properdin functionality.

In addition, using the Salp20 method we observed that convertase assembly, especially in absence of properdin, depended on C3 levels (Figure 6). Addition of purified C3 to serum samples with low C3 levels could shift the delayed t_max_ to earlier time points and restore the ability to analyse the samples for convertase stabilization (Figures 5, 6, 8). Interestingly, the samples of P5, P7, P9, and to a lesser extent P2, also had C3 levels below the normal range but did not require C3 supplementation. The potency of serum to support efficient convertase formation depends not only on C3 concentration but also on other complement (regulatory) proteins (and possibly C3NeFs), which may altogether give favorable profiles even in combination with low C3 in some patients. Nevertheless, in general, the patients who did require C3 supplementation (P1, P3, P4, P8, P10, P11, and P13) all have low C3 levels, whereas the only ones with C3 levels within the normal range (P6 and P12) did not require C3 supplementation for analysis.

Previous studies have shown that Salp20 displaces properdin from the convertase due to the higher affinity of properdin for Salp20 than for C3b (45, 46). In our hands, Salp20 was indeed an effective inhibitor of properdin (Figure 2). It has been shown that Salp20 is highly specific for properdin inhibition (45, 46). No other functions of Salp20 have been reported, and we did not encounter any critical side effects of Salp20 in current assay settings. Though, we cannot explain why high Salp20 concentrations partially inhibited maximal hemolysis levels (Figure 2C).

Using Salp20 as a model, we here evaluated the potential of properdin-inhibiting therapy in patients with properdin-dependent C3NeFs. Salp20 could effectively block properdin and normalize the prolonged convertase activity caused by properdin-dependent C3NeFs in P4, P10, and P13 (Figures 5, 6, 8). In patients with properdin-independent C3NeFs, properdin inhibition did not affect the increased convertase stability. These results support that properdin inhibition has a therapeutic potential for patients with properdin-dependent C3NeFs, but not for patients with properdin-independent C3NeFs. For the latter, alternative therapeutic strategies are needed, e.g., inhibition at the level of C3 in other ways.

Blocking properdin seems a relatively safe approach for humans, since properdin-deficient individuals do not show a severely compromised immune function, apart from the increased susceptibility to meningitis for which vaccination is possible (47, 48). Few studies have described blocking anti-human properdin antibodies or human properdin-competing compounds so far, and some were used in *in vitro* and *ex vivo* complement-mediated disease models (49–54). Nevertheless, therapy directed at blocking properdin must be considered with care, since studies in mice have shown that inhibition of properdin might result in unexpected outcomes (20). In two separate C3G mouse models, it was shown that blocking properdin exacerbated instead of improved disease outcome (55, 56). However, these models were not initiated by C3NeF activity but by absence of functional FH, a cause which only occurs in rare cases of human C3G. Thus, upon properdin-inhibition, the initial cause of the overactive complement activity, namely dysfunctional FH, still remained present. In individuals in which properdin-dependent C3NeFs are responsible for the complement overactivity, blockage of properdin tackles the causing factor (albeit indirectly). Further *in vivo* experiments using human-compatible properdin blockers are needed to investigate the therapeutic potential of properdin inhibition in C3G patients. Potentially, temporary therapy with a properdin inhibitor might already be enough to bring patients back to a state of normal complement homeostasis.

In conclusion, we here demonstrated two simple, time-effective, and reliable methods to distinguish between properdin-dependent and properdin-independent C3NeFs. We also showed that properdin inhibition in patients with properdin-dependent C3NeFs may be a viable therapeutic strategy to normalize convertase activity.

## Ethics Statement

This study was carried out in accordance with the recommendations of the appropriate version of the Declaration of Helsinki. All subjects gave written informed consent, and the protocol was approved by the ethical committee of the Radboud University Medical Center (FH 06979).

## Author Contributions

Concept of the study: MM, EV, LvdH, and NvdK. Design of the experiments: MM, EV, and LvdH. Experimental work: MM, TvdV, SvK, SS, RvdB. Data analysis: MM, NvdK, RvdB, EV, and LvdH. Collection and characterization of clinical samples (including genetic data): MM, NvdK, VG, MO, EV, and LvdH. Manuscript writing: MM. All the authors approved the manuscript.

### Conflict of Interest Statement

NvdK is a member of the international aHUS Advisory Board of Alexion. The remaining authors declare that the research was conducted in the absence of any commercial or financial relationships that could be construed as a potential conflict of interest.

## Abbreviations

AP: Alternative pathway
C3G: C3 glomerulopathy
C3GN: C3 glomerulonephritis
C3NeF(s): C3 nephritic factor(s)
DDD: Dense deposit disease
EDTA: Ethylenediamine-tetraacetic acid
ELISA: Enzyme-linked immunosorbent assay
hi-NHS: Heat-inactivated pooled normal human serum
Ig(s): Immunoglobulin(s)
FH: Factor H
FI: Factor I
IC-MPGN: Immune complex-mediated membranoproliferative glomerulonephritis
Mg-EGTA: Magnesium-ethylene glycol tetraacetic acid
NHP: Pooled normal human EDTA-plasma
NHS: Pooled normal human serum
RbE: Rabbit erythrocyte
t_max_: (time point of) maximal convertase activity
ΔP-NHS: Properdin-depleted normal human serum
ΔC3-NHS: C3-depleted normal human serum.
