# Supplementary material for: Novel Assays to Distinguish Between Properdin-Dependent and Properdin-Independent C3 Nephritic Factors Provide Insight Into Properdin-Inhibiting Therapy
Source: Front Immunol. 2019 Jun 17;10:1350. doi: 10.3389/fimmu.2019.01350 (PMC6590259; doi:10.3389/fimmu.2019.01350)
Supplement: Supplementary file 1 [file Presentation_1.PPTX]

## Slide 1
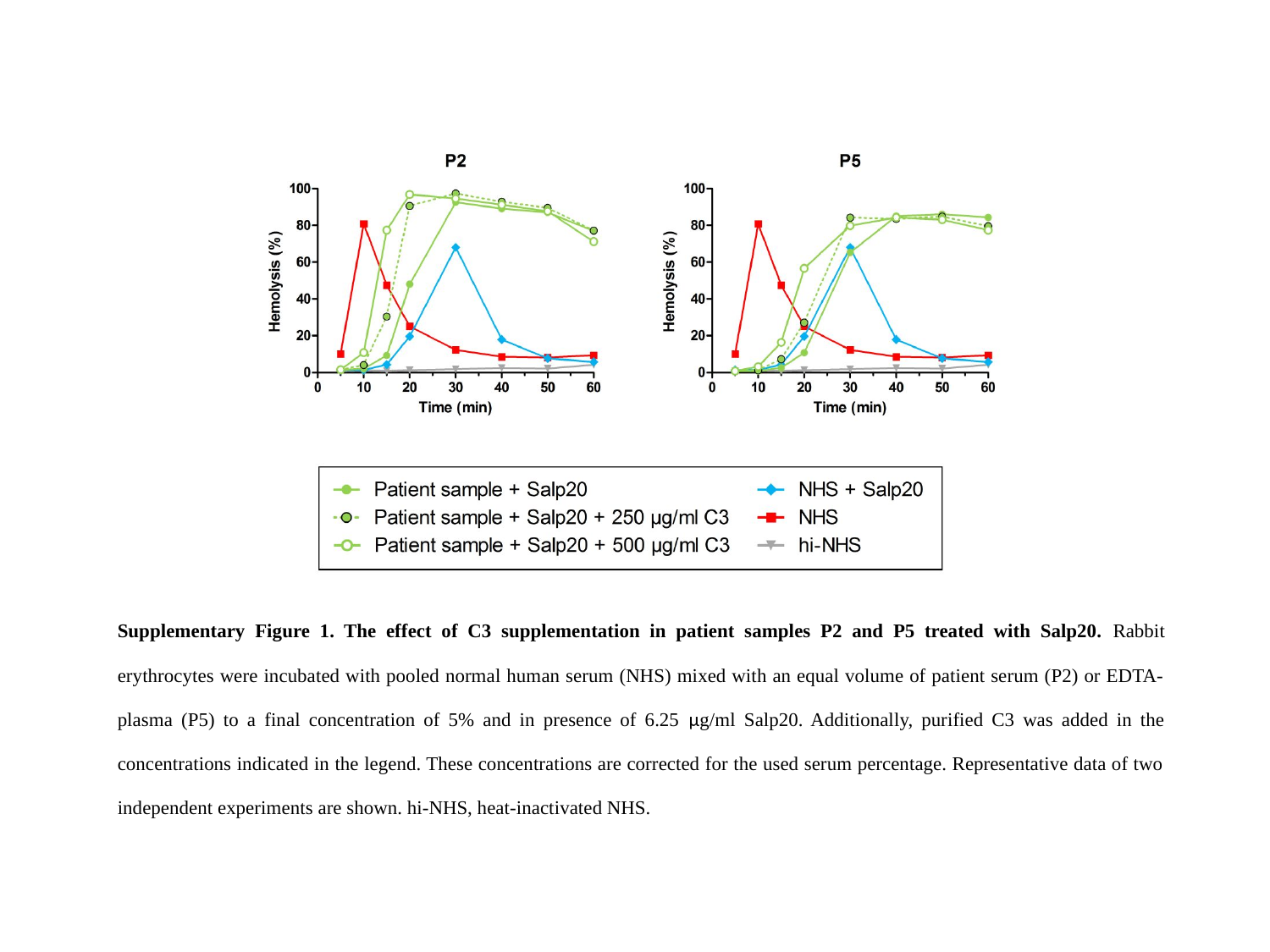

Supplementary Figure 1. The effect of C3 supplementation in patient samples P2 and P5 treated with Salp20. Rabbit erythrocytes were incubated with pooled normal human serum (NHS) mixed with an equal volume of patient serum (P2) or EDTA-plasma (P5) to a final concentration of 5% and in presence of 6.25 µg/ml Salp20. Additionally, purified C3 was added in the concentrations indicated in the legend. These concentrations are corrected for the used serum percentage. Representative data of two independent experiments are shown. hi-NHS, heat-inactivated NHS.
